# Head-to-head comparison of tau positron emission tomography tracers [^18^F]flortaucipir and [^18^F]RO948

**DOI:** 10.1007/s00259-019-04496-0

**Published:** 2019-10-14

**Authors:** Ruben Smith, Michael Schöll, Antoine Leuzy, Jonas Jögi, Tomas Ohlsson, Olof Strandberg, Oskar Hansson

**Affiliations:** 1 Clinical Memory Research Unit, Department of Clinical Sciences, Lund University, Malmö, Sweden; 2 Department of Neurology, Skåne University Hospital, Lund, Sweden; 3 Department of Neurology, Skåne University Hospital, SE-20502 Malmö, Sweden; 4 Wallenberg Centre for Molecular and Translational Medicine and the Department of Psychiatry and Neurochemistry, University of Gothenburg, Gothenburg, Sweden; 5 Dementia Research Centre, Department of Neurodegenerative Disease, Queen Square Institute of Neurology, University College London, London, UK; 6 Department of Clinical Physiology and Nuclear Medicine, Skåne University Hospital, Lund, Sweden; 7 Department of Radiation Physics, Skåne University Hospital, Lund, Sweden; 8 Memory Clinic, Skåne University Hospital, SE-20502 Malmö, Sweden

**Keywords:** Tau, PET, Neurodegeneration, Alzheimer’s disease, Head-to-head

## Abstract

**Purpose:**

[^18^F]flortaucipir binds to paired helical filament tau and accurately identifies tau in Alzheimer’s disease (AD). However, “off-target” binding interferes with the quantification of [^18^F]flortaucipir in several brain regions. Recently, other tau PET tracers have been developed. Here, we compare [^18^F]flortaucipir with the novel tau tracer [^18^F]RO948 head-to-head in vivo.

**Methods:**

We included 18 participants with AD, three with amyloid-β-positive amnestic mild cognitive impairment, and four healthy controls. All underwent [^18^F]flortaucipir (80–100 min) and [^18^F]RO948 (70–90) PET scans within approximately 1 month. Four study participants underwent 0–100-min dynamic scanning. Standardized uptake value ratios (SUVRs) were created using an inferior cerebellar reference region.

**Results:**

Neocortical tracer retention was highly comparable using both SUVR and distribution volume ratio-1 values obtained from dynamic scans. However, [^18^F]RO948 retention was significantly higher in the entorhinal cortex and lower in the basal ganglia, thalamus, and choroid plexus compared with [^18^F]flortaucipir. Increased off-target binding was observed with age for both tracers. Several cases exhibited strong [^18^F]RO948 retention in the skull/meninges. This extra-cerebral signal, however, did not affect diagnostic accuracy and remained relatively unchanged when re-examining a subsample after 1 year. Kinetic modeling showed an increase in [^18^F]flortaucipir SUVR over the scanning interval, compared with a plateau for [^18^F]RO948.

**Conclusion:**

[^18^F]RO948 and [^18^F]flortaucipir bound comparably in neocortical regions, but [^18^F]RO948 showed higher retention in the medial temporal lobe and lower intracerebral “off-target” binding. Time-dependent bias of SUVR estimates may prove less of a factor with [^18^F]RO948, compared with previous tau ligands.

**Electronic supplementary material:**

The online version of this article (10.1007/s00259-019-04496-0) contains supplementary material, which is available to authorized users.

## Introduction

Alzheimer’s disease is characterized neuropathologically by the gradual deposition of amyloid-β (Aβ) into senile plaques and the aggregation of hyperphosphorylated tau into neurites and intrasomal neurofibrillary tangles [1, 2]. The tau pathology associated with Alzheimer’s disease mainly consists of paired helical filaments (PHFs), formed by a mixture of three- and four-repeat tau isoforms [3]. This tau pathology is closely related to measures of neurodegeneration such as decreased glucose metabolism [4, 5] and to the development of cognitive symptoms [6–8].

During the past decade, a number of positron emission tomography (PET) radiotracers have been developed for mapping the deposition of tau pathology in vivo [9–17]. The hitherto most used and best-validated tracer is [^18^F]flortaucipir (previously known as [^18^F]-AV-1451 and [^18^F]-T807). Using autoradiography, [^18^F]flortaucipir has been shown to bind strongly to tau pathology in post mortem Alzheimer’s disease brain tissue [18–20]. In vivo*,* [^18^F]flortaucipir retention has been shown to correlate well with post mortem tau pathology in *MAPT R406W* mutation gene carriers [21] and to Alzheimer’s disease-related tau pathology [22]. In addition, [^18^F]flortaucipir performs excellently in distinguishing AD from other neurodegenerative disorders based on retention of the tracer in the temporal cortex [23].

However, [^18^F]flortaucipir also shows substantial retention in the basal ganglia, the thalamus, and the choroid plexus, where no PHF tau pathology is expected [18–20], likely representing “off-target” binding. Novel tau PET tracers such as [^18^F]RO948, [^18^F]PI-2620, [^18^F]GTP1, and [^18^F]MK-6240 have slightly different binding properties compared with [^18^F]flortaucipir [9, 10, 15]. However, no study has yet compared the binding characteristics of [^18^F]flortaucipir with a “second generation” tau tracer in vivo in the same individuals. We therefore performed a head-to-head comparison of [^18^F]flortaucipir and [^18^F]RO948 in patients with Alzheimer’s disease, mild cognitive impairment due to Alzheimer’s disease, and cognitively healthy controls. We assessed the degree and regional distribution of cortical tracer retention of [^18^F]flortaucipir and [^18^F]RO948, as well as whether the radiotracers exhibit differences in the distribution and intensity of “off-target” binding.

## Methods

### Participants

For the head-to-head study, we included 18 patients with AD dementia who fulfilled the Diagnostic and Statistical Manual of Mental Disorders (Fifth Edition; DSM-V) criteria for probable AD [24], three patients with amnestic mild cognitive impairment (MCI-AD) [25, 26], and four neurologically healthy controls. Patients with MCI-AD exhibited objective memory impairment, MMSE scores between 24 and 30, and low CSF Aβ_42_/Aβ_40_, and did not fulfill criteria for dementia. Inclusion criteria for neurologically healthy controls were (a) aged 40–100 years, (b) 26–30 points at Mini-Mental State Examination (MMSE) at the screening visit, (c) absence of cognitive symptoms as assessed by a physician with special interest in cognitive disorders, (d) subjects that did not fulfill criteria for MCI or any dementia and according to DSM-V [24], and (e) fluency in Swedish. Exclusion criteria for all participants were (a) significant systemic illness hindering participation, (b) significant neurologic or psychiatric disease other than the inclusion disorder, (c) alcohol or substance abuse, or (d) refusing lumbar puncture, MRI, or PET. For demographics, see Table 1.

**Table 1:** Clinical characteristics and demographics

	AD	MCI-AD	Controls	*P* value
*n*	18	3	4	
Age (y)	73.1 ± 7.4	77.3 ± 4.0	64.5 ± 10.9	n.s.
Sex (M/F)	11/7	0/3	2/2	AD vs MCI *P* < 0.05
Education (y)	13.8 ± 5.8	13 ± 8.7	12.5 ± 3.4	n.s.
MMSE	22.2 ± 3.9	27.6 ± 0.6	29.3 ± 0.6	AD vs Controls *P* < 0.01; AD vs MCI *P* < 0.01
Amyloid status (% positive)	100	100	50	AD vs controls *P* < 0.01
ApoE4 (0/+/++)	6/11/1	1/2/0	3/1/0	n.s.

*n.s.* non significant

In order to further examine the effect of age on off-target binding in the basal ganglia, we further included individuals from the BioFINDER1 (www.biofinder.se) study who had undergone examination with [^18^F]flortaucipir (*n* = 212; inclusion criteria for the different diseases and controls are described elsewhere [27–30]) and individuals from the BioFINDER2 study who had been examined with [^18^F]RO948 (*n* = 465). The BioFINDER2 study enrolls participants in five sub-cohorts. Cohorts A and B include neurologically and cognitively healthy controls. The inclusion criteria are (i) ages 40–65 years (cohort A) and ages 66–100 years (cohort B); (ii) absence of cognitive symptoms as assessed by a physician with special interest in cognitive disorders; **(**iii) MMSE score 27–30 (cohort A) or 26–30 (cohort B) at screening visit; and (iv) those who do not fulfill the criteria for MCI or any dementia according to DSM-V [24]. Cohort C comprises participants with subjective cognitive deficits (SCD), or minor neurocognitive impairment (MCI) (the latter according to DSM-5 [24]). Inclusion criteria are (i) age 40–100 years; (ii) those referred to the memory clinics due to cognitive symptoms; (iii) MMSE score of 24–30 points; and (iv) those who do not fulfill the criteria for any dementia (major neurocognitive disorder) according to DSM-5 [24]. Cohort D consists of participants with dementia due to AD. Inclusion criteria are (i) age 40–100 years; (ii) those referred to the memory clinics due to cognitive symptoms; (iii) MMSE score of ≥ 12 points; and (iv) those who fulfill the DSM-5 criteria for dementia (major neurocognitive disorder) due to Alzheimer’s disease [24]. Cohort E covers other non-AD dementias and neurodegenerative disorders. Inclusion criteria are (i) age 40–100 years and (ii) fulfillment of criteria for dementia (major neurocognitive disorder) due to frontotemporal dementia [24], Parkinson’s disease (PD) with dementia [24], dementia with Lewy bodies [24] or subcortical vascular dementia [24] alternatively the criteria for PD [31], progressive supranuclear palsy [32], multiple system atrophy [33], corticobasal syndrome [34], or semantic variant primary progressive aphasia [35]. Included participants in all cohorts had to be fluent in Swedish. Exclusion criteria for all sub-cohorts are (i) significant unstable systemic illness that makes it difficult to participate in the study; (ii) current significant alcohol or substance misuse; (iii) refusing lumbar puncture, MRI, or PET.

All participants gave written informed consent to participate in the study. The informed consent forms signed by AD patients were also signed by their informant. Potential participants judged by the treating physician not able to give informed consent due to advanced dementia were not included in the study. Ethical approval was obtained from the Regional ethics committee at Lund University, Sweden. All imaging procedures were approved by the Radiation protection committee at Skåne University Hospital and by the Swedish Medical Products Agency.

### MR imaging

All participants underwent 3.0 T MRI scans (Siemens MAGNETOM Prisma), acquiring isometric 1 mm^3^ T1-weighted magnetization-prepared rapid gradient-echo and fluid-attenuated inversion recovery images. MR images were processed using an in-house-developed pipeline including the removal of non-brain tissue (brain extraction), segmentation into grey and white matter, parcellation into regions of interest (ROI), and normalization of images into Montreal Neurological Institute (MNI152) standard space.

### PET imaging

All study participants underwent two PET scans on a digital GE Discovery MI scanner (General Electric Medical Systems), with an average of 36 ± 35 days between scans. Participants were injected with 341 ± 53 MBq of [^18^F]flortaucipir or 365 ± 20 MBq of [^18^F]RO948, and LIST mode emission data was acquired for each scan of 80–100 min ([^18^F]flortaucipir) or 70–90 min ([^18^F]RO948) post injection. Different time frames for image acquisition were chosen due to different pharmacokinetics as described previously [15, 36].

Low-dose CT scans were performed immediately prior to the PET scans for attenuation correction. PET data was reconstructed using VPFX-S (ordered subset expectation maximization (OSEM) with time-of-flight (TOF) and point spread function (PSF) corrections) with 6 iterations and 17 subsets with 3 mm smoothing, standard Z filter, and 25.6-cm field of view with a 256 × 256 matrix. LIST mode data was binned into 4 × 5-min time frames, and the resulting PET images motion corrected, summed, and co-registered to their corresponding T1-weighted MR images.

### Image data processing and analysis

ROIs were based on the parcellation of the T1-weighted MRI using FreeSurfer v6.0 (https://surfer.nmr.mgh.harvard.edu/). Standardized uptake value ratio (SUVR) images were calculated using an inferior cerebellar reference region [37]. For comparison, SUVR images were also created using the whole cerebellum and an eroded white matter reference region. Partial volume correction (PVC) was performed using the geometric transfer matrix method [38]; both corrected and uncorrected data were analyzed. Composite ROIs were created for regions corresponding to image-based tau stages, as defined in [39]—I/II (entorhinal), III/IV (temporal/limbic), V/VI (neocortical), and I–IV (temporal meta-ROI)—and the basal ganglia (caudate nucleus, putamen, and globus pallidus).

For voxelwise analyses, SUVR PET images were spatially transformed into a common MNI152 space using the transformation derived from MRI normalization and smoothed at 6 mm with a full width at half maximum Gaussian kernel. Calculations were performed using SPM12 (Wellcome Department of Cognitive Neurology, London, UK; http://www.fil.ion.ucl.ac.uk/spm) in MATLAB (v. 9.2, 2017a).

To quantify tracer uptake in the skull and the meninges in the individuals recruited to this study, an ROI was created using an eroded binarized combination of bone and soft tissue masks derived from the SPM12 tissue segmentations of each individual’s T1-weighted MRI. Further, [^18^F]RO948 retention in the skull and the meninges was assessed for further 538 subjects from BioFINDER2 (scale 0.5–3 SUVR) by an assessor blinded to clinical information, with retention in these regions rated as “normal”/“minor” (no or only limited regions within the skull showing a low grade retention), “moderate” (more confluent areas of moderate retention or smaller areas with high retention), or “high” (confluent areas of high retention (SUVR > 2.5; red upon visual inspection)). A receiver operator characteristic analysis was performed for the diagnostic performance in separating AD from controls with and without subjects with high off-target retention in the skull using the pROC package in R v3.4.

Data used for the basal ganglia vs age correlation analysis in the whole BioFINDER1 and BioFINDER2 tau PET cohorts (see below and Fig. 3d) were acquired on a GE Discovery 690 PET/CT camera, 80–100 min after injection of ~ 370 MBq [^18^F]flortaucipir (BioFINDER1), and on digital GE Discovery MI scanners 70–90 min after injection of ~ 370 MBq [^18^F]RO948 (BioFINDER2).

### Kinetic modeling

Four subjects (all Alzheimer’s disease dementia patients) underwent dynamic PET scanning at 0–100 min post tracer injection for both [^18^F]flortaucipir and [^18^F]RO948. The data was acquired in LIST mode and was reconstructed into 48 time frames (12 × 10 s, 6 × 20 s, 6 × 30 s, 3 × 60 s, 5 × 120 s, and 16 × 300 s). The dynamic images were processed in the same way as for the shorter acquisitions, with time-activity curves (TACs) extracted from all ROIs. Kinetic modeling was performed ROI-wise using a MATLAB-based implementation of the Logan reference model [40] over the time interval 30–80 min, using the inferior cerebellar cortex as the reference region. Voxelwise implementation of this model was performed using PMOD (v.3.7, PMOD Technologies Ltd., Zurich, Switzerland).

### Statistics

Statistical analyses for ROI-based comparisons were performed using R v3.4 or GraphPad Prism 7 for Mac. Group comparisons were performed using Wilcoxon’s signed-rank tests and Spearman’s rank correlation (*rho*). Statistical significance was assumed at *P* < 0.05, adjusted for multiple comparisons using Bonferroni correction. For the voxelwise comparison of [^18^F]flortaucipir and [^18^F]RO948 retention patterns, we employed a voxelwise paired two-sample *t* test, as implemented in SPM12, using the time between examinations as a covariate, masking the resulting t-maps with a brain mask in MNI space. Resulting t-statistics were thresholded at a significance level of *P* < 0.05 (corrected for family-wise error (FWE)) and a cluster size of *k* > 50.

## Results

### Kinetic modeling

**Fig. 1:**
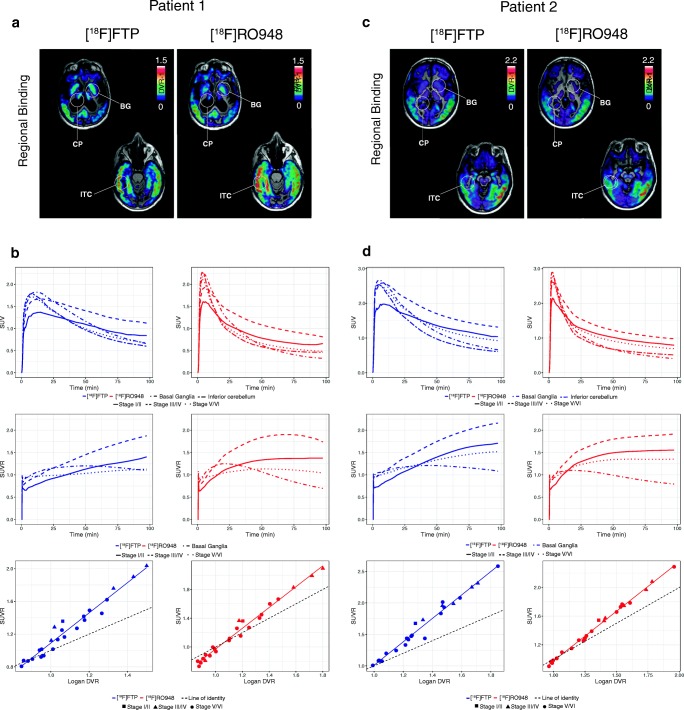
Comparison of [^18^F]flortaucipir (FTP) and [^18^F]RO948 parametric BP_ND_ (Logan DVR-1) images (**a**, **c**) and regional SUV, SUVR, and SUVR vs DVR data (**b, d**) in two cases with AD dementia with high tau burden. Stage I/II, entorhinal cortex; stage III/IV, temporal/limbic cortex; stage V/VI, neocortex. CP, choroid plexus; BG, basal ganglia; FTP, Flortaucipir; ITC, inferior temporal cortex

**Fig. 2:**
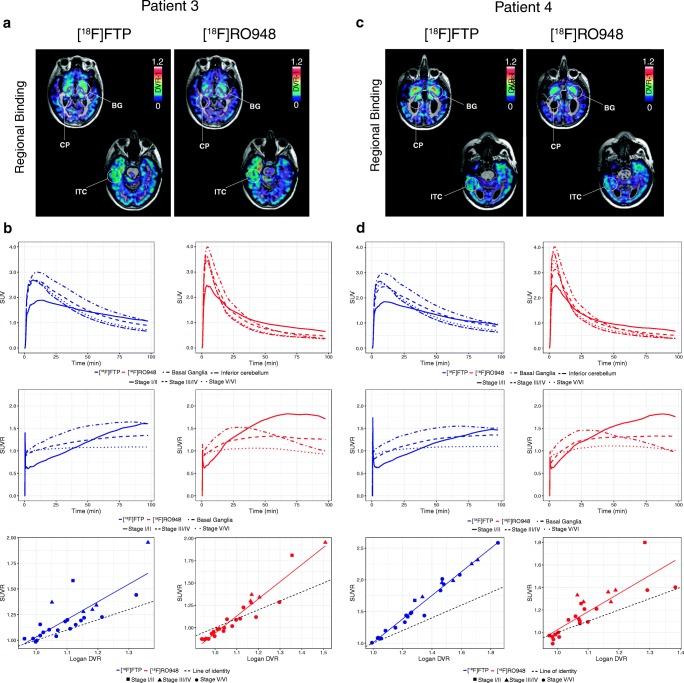
Comparison of [^18^F]flortaucipir (FTP) and [^18^F]RO948 parametric BP_ND_ (Logan DVR-1) images (**a**, **c**) and regional SUV, SUVR, and SUVR vs DVR data (**b, d**) in two cases with AD dementia with more limited tau burden. Stage I/II, entorhinal cortex; stage III/IV, temporal/limbic cortex; stage V/VI, neocortex. CP, choroid plexus; BG, basal ganglia; FTP, Flortaucipir; ITC, inferior temporal cortex

First, we analyzed the fully dynamic scans (0–100 min) in the four Alzheimer’s disease dementia cases. Comparison of TACs showed faster kinetics for [^18^F]RO948 (two cases are depicted in Figs. 1 and 2). When examining SUVRs over the full 100-min interval, SUVR values for [^18^F]flortaucipir appeared to continuously increase over time, in contrast to those for [^18^F]RO948, which reached a plateau during the scanning period.

In general, similar binding patterns were found for both tracers in neocortical regions (Figs. 1 and 2). Though not absent, the signal from the basal ganglia, the thalamus, and the choroid plexus appeared lower on [^18^F]RO948 scans than that on [^18^F]flortaucipir scans. Strong correlations were observed between regional SUVR and DVR data for both tracers in the two patients depicted in Fig. 1 exhibiting more substantial tau pathology ([^18^F]flortaucipir, *R*^2^ = 0.978 and 0.988, *P* < 0.001; [^18^F]RO948, *R*^2^ = 0.991 and 0.998, *P* < 0.001). In the two cases with more limited tau pathology, we found more modest correlations (Fig. 2; [^18^F]flortaucipir, *R*^2^ = 0.800 and *R*^2^ = 0.702, *P* < 0.001; [^18^F]RO948, *R*^2^ = 0.960 and *R*^2^ = 0.840, *P* < 0.001). SUVR values tended to overestimate DVR values (slopes of both [^18^F]flortaucipir and [^18^F]RO948 were significantly different from the line of origin) particularly when examining high DVR values. While this appeared more pronounced for [^18^F]flortaucipir, the slopes of the fits for both tracers were not significantly different.

### Cortical retention of radiotracers

**Fig. 3:**
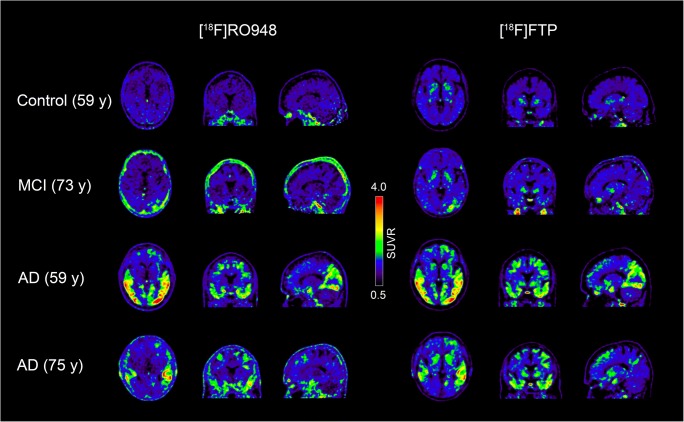
Representative [^18^F]flortaucipir (FTP) and [^18^F]RO948 images for two patients with AD dementia, one patient with MCI due to AD, and one cognitively healthy individual

Next, we compared 20-min (4 × 5 min) scans in 25 participants who had been enrolled in the head-to-head comparison study. The clinical and demographic characteristics of the participants are presented in Table 1. Representative [^18^F]flortaucipir and [^18^F]RO948 images of four cases are presented in Fig. 3.

**Fig. 4:**
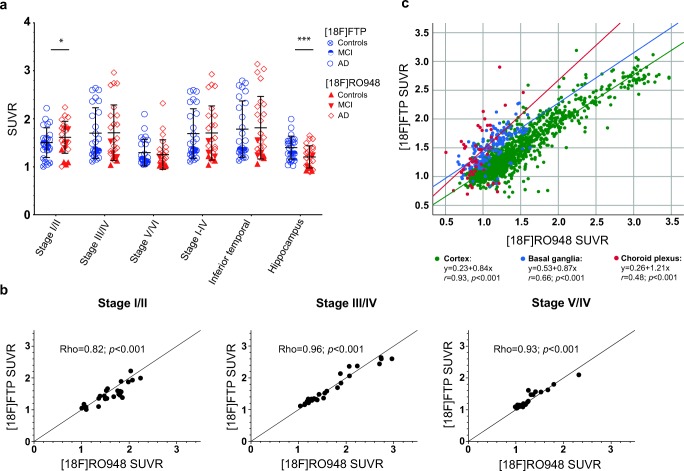
Comparison of SUVRs of [^18^F]flortaucipir (FTP) and [^18^F]RO948 in selected cortical ROIs (**a**). Correlations of [^18^F]flortaucipir and [^18^F]RO948 retentions in composite ROIs corresponding to regions of Braak stages (**b**). Scatter plot of all FreeSurfer-derived cortical, basal ganglia, and choroid plexus ROIs for both tracers (**c**). Braak imaging: stage I/II, entorhinal cortex; stage III/IV, temporal/limbic cortex; stage V/VI, neocortex; I–IV, temporal meta-ROI

When evaluating ROI-based SUVRs, retention was comparable using both radiotracers in the composite regions corresponding to tau imaging stages III/IV (temporal/limbic), V/VI (neocortical), and I–IV (temporal “meta-ROI”), as well as in the inferior temporal cortex (Fig. 4a). We found slightly higher retention of [^18^F]RO948 in the entorhinal cortex (stage I/II) compared with [^18^F]flortaucipir (1.62 ± 0.33 vs 1.50 ± 0.30, *P* < 0.05; Fig. 4a). On the other hand, [^18^F]flortaucipir retention was higher in the hippocampus (Fig. 4a). This hippocampal [^18^F]flortaucipir binding was significantly correlated with retention in the adjacent choroid plexus (Spearman’s rho = 0.37, *P* = 0.05), which was not the case for [^18^F]RO948 (rho = 0.2, *P* = 0.34). The association became less pronounced after PVC ([^18^F]flortaucipir: Rho = 0.15*, P =* 0.05; [^18^F]RO948: rho = 0.01, *P* = 0.98) suggesting that at least a proportion of hippocampal [^18^F]flortaucipir uptake could be attributed to off-target binding in the choroid plexus.

As depicted in Fig. 4b and c, retention of the two tracers was highly correlated in ROIs corresponding to stages I/II (0.83; *P* < 0.001), III/IV (0.97; *P* < 0.001), and V/VI (0.94; *P <* 0.001) (Fig. 4b) as well as in all cortical ROIs (*r* = 0.93, *P <* 0.001) (Fig. 4c). The slope of 0.84 ([^18^F]flortaucipir-dependent variable) for cortical ROIs suggested a slightly higher dynamic range for [^18^F]RO948 in the cortex (Fig. 4c). Similar results were obtained using PVC data (stage I/II (0.74; *P* < 0.001), III/IV (0.93; *P* < 0.001), and V/VI (0.92; *P <* 0.001)).

Finally, we compared the difference in SUVR between the two tracers using a test-retest approach for pre- vs post-PVC, separately, and for different reference regions employed. Comparison of SUVR between PVC and non-PVC ROI-data showed highly similar values throughout cortex; only the difference in retention for the two tracers in the hippocampus was decreased post-PVC (Table 2). The use of different reference regions also yielded similar SUVR across cortical regions for both tracers (Table 2).

**Table 2:** Comparison of differences in SUVR ([18F]RO948 vs [18F]flortaucipir) pre and post partial volume correction and using different reference regions

	Non-PVC mean (% difference^§^)	PVC mean (% difference^§^)	Inferior CBL mean (% difference^§^)	Whole CBL mean (% difference^§^)	Eroded WhM mean (% difference^§^)
Stage I/II	7.85	6.96	6.96	9.33	5.93
Stage III/IV	− 0.28	− 1.06	− 0.28	2.08	− 1.35
Stage V/VI	− 3.58	− 4.24	− 3.58	− 1.22	− 4.69
Stage I–IV	0.14	− 0.50	0.14	2.50	− 0.92
Inferior temporal ctx	0.76	0.32	0.76	3.12	− 0.31
Hippocampus	− 19.83	− 15.09	− 15.09	− 12.75	− 16.24

Stage I/II corresponds to entorhinal cortex, stage III/IV temporal/limbic cortex, stage V/VI neocortex, and I–IV corresponds to a temporal meta-ROI.

*CBL*, cerebellum; *ctx*, cortex; *PVC*, partial volume error correction; *WhM*, white matter

^§^Formula used to calculate differences (mean in percent) between regional tracer retention: 100 × ([18F]RO948 SUVR – [18F]flortaucipir SUVR)/(([18F]RO948 SUVR + [18F]flortaucipir SUVR)/2

### “Off-target” binding

**Fig. 5:**
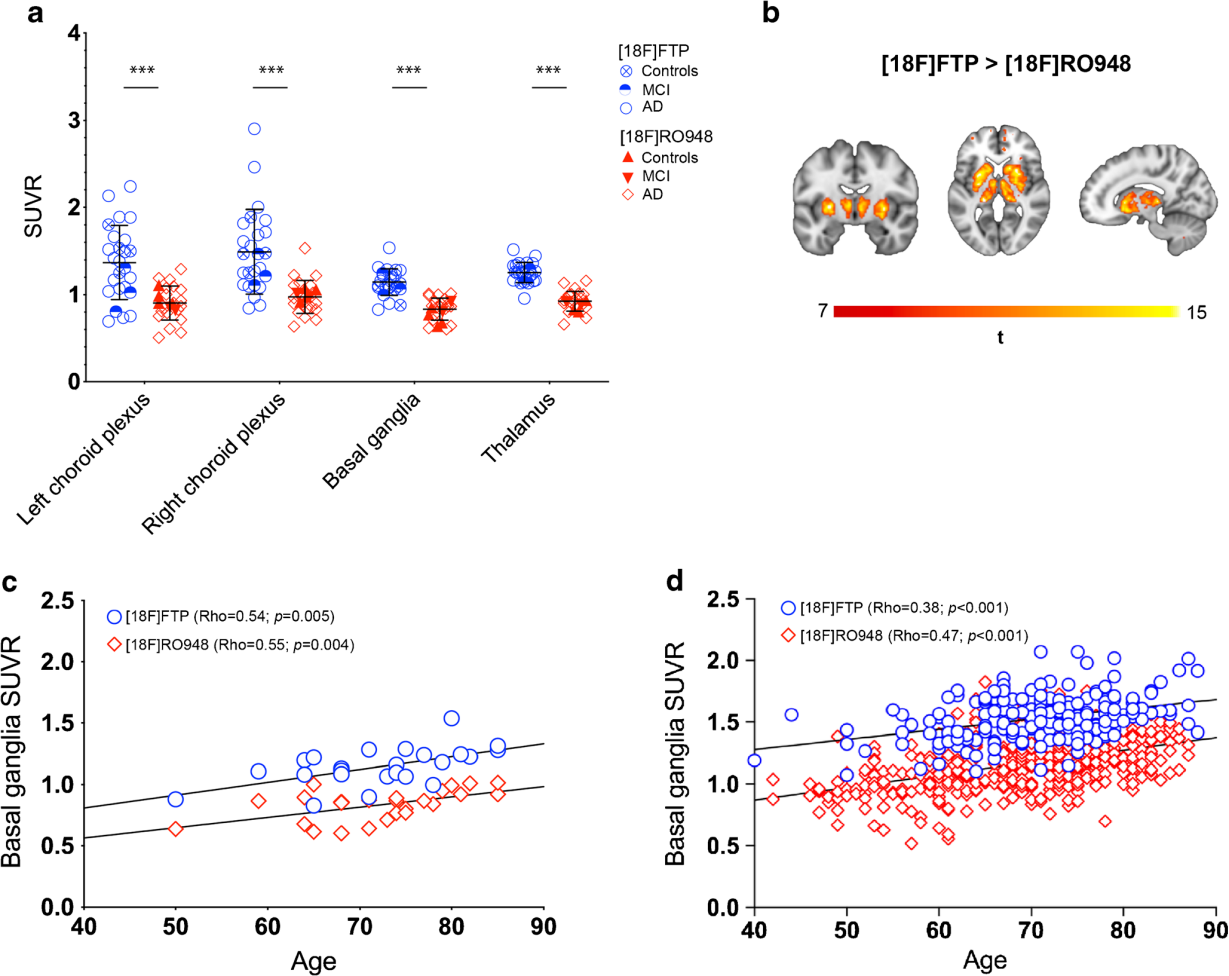
Comparison of SUVR of [^18^F]flortaucipir (FTP) and [^18^F]RO948 in ROIs covering typical sites of [^18^F]flortaucipir off-target binding regions (**a**). Voxelwise comparison where [^18^F]flortaucipir exhibited higher retention than [^18^F]RO948 and yielded significant voxels in the striatum and the thalamus (**b**). Association of tracer retention in the basal ganglia with age in this study’s sample (*n* = 25; some data points are hidden behind other data points) (**c**) and in the whole BioFINDER 1 cohort ([^18^F]flortaucipir; *n* = 212) and BioFINDER 2 cohort ([^18^F]RO948 *n* = 465) (**d**)

When comparing tracer binding in the classical “off-target” regions for [^18^F]flortaucipir, i.e., the basal ganglia, thalamus, and the choroid plexus, [^18^F]flortaucipir retention was significantly higher compared with [^18^F]RO948 (basal ganglia 1.15 ± 0.15 vs 0.83 ± 0.13, *P <* 0.001; thalamus 1.26 ± 0.12 vs 0.94 ± 0.11, *P <* 0.001; choroid plexus 1.43 ± 0.43 vs 0.94 ± 0.19, *P <* 0.001; Fig. 5a). Voxelwise analysis using a statistical threshold at *P <* 0.05 (corrected for FWE) and a cluster size of *k* > 50 voxels confirmed significantly increased [^18^F]flortaucipir retention in the basal ganglia and thalamus compared with [^18^F]RO948 (Fig. 5b). The difference in the signal in the choroid plexus did not yield statistical significance, likely due to the substantial anatomical variability of this structure. No significant clusters were found when using the reverse contrast ([^18^F]RO948 > [^18^F]flortaucipir). We next compared the association between basal ganglia retention and age for [^18^F]RO948 and [^18^F]flortaucipir within this study (Fig. 5c) and the same association for [^18^F]flortaucipir within the BioFINDER1 (BF1) cohort (*n* = 212) and for [^18^F]RO948 within BioFINDER2 (BF2) cohort (*n* = 465). The slopes were comparable (BF1: [^18^F]flortaucipir, 0.008 [95% CI, 0.005–0.011]; BF2: [^18^F]RO948, 0.010 [95% CI, 0.009–0.012]) but the Y-intercept was clearly higher for [^18^F]flortaucipir (0.96 [95% CI 0.76–1.15]; [^18^F]RO948, 0.47 [95% CI, 0.37–0.58]; Fig. 5d), indicating the overall higher levels of [^18^F]flortaucipir in these regions.

**Fig. 6:**
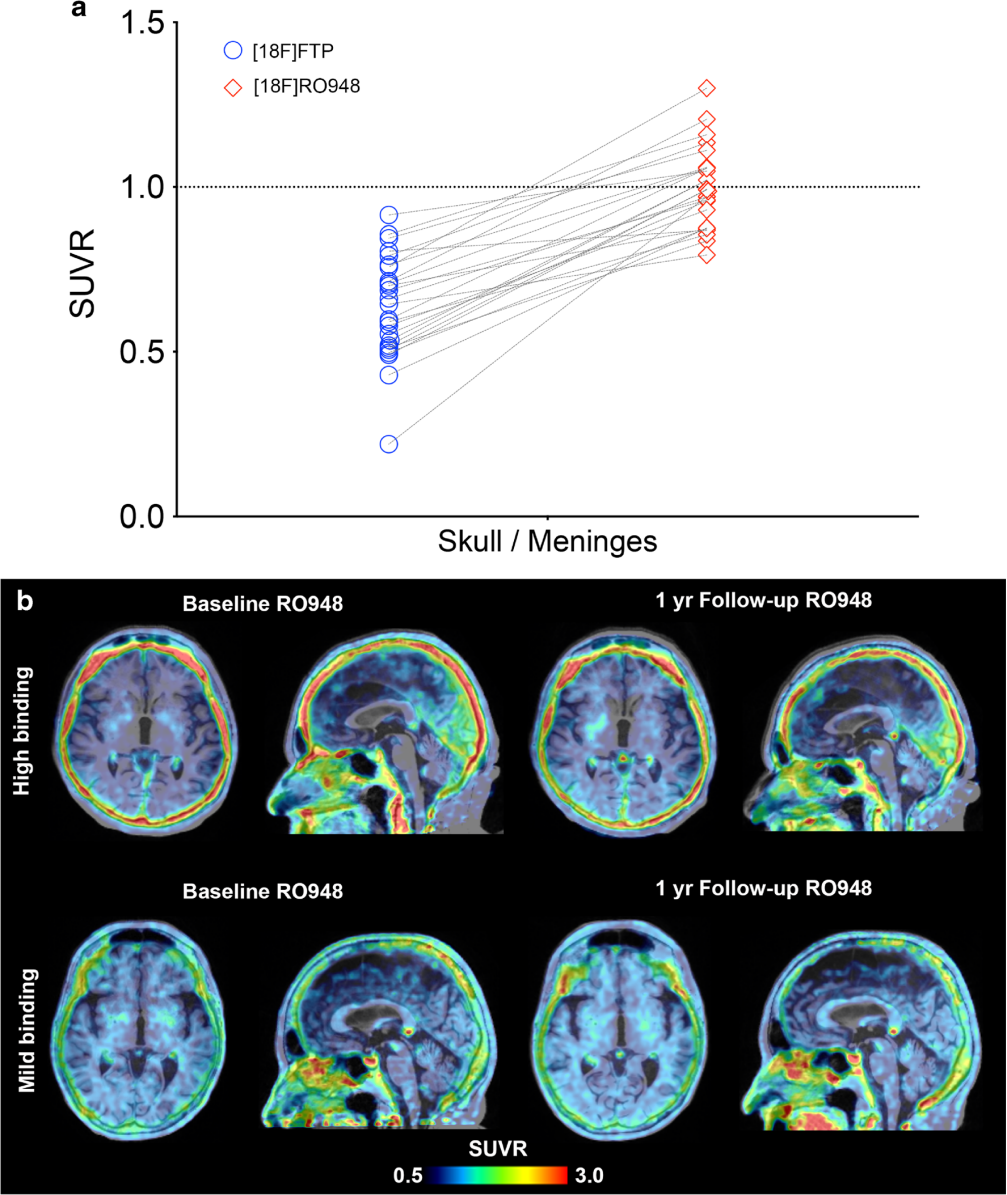
Quantification of [^18^F]RO948 and [^18^F]flortaucipir (FTP) uptake in an ROI covering the skull bone and the meninges (**a**). Comparison between [^18^F]RO948 retention at baseline and after 1 year (**b**)

In contrast, [^18^F]RO948 showed higher retention in the skull and meninges compared with [^18^F]flortaucipir (0.64 ± 0.16 vs 0.99 ± 0.12, *P <* 0.001; Fig. 6a and Fig. 3). Visual assessment of this off-target binding within the BioFINDER2 cohort indicated high skull/meningeal retention in 3.4% of subjects and moderate uptake in 9.3% of all study participants. Removing individuals with high skull/meningeal retention did not result in increased area under the curve in receiver operator characteristic curves when comparing AD patients with controls in tau imaging stages I/II or I–IV, suggesting that this off-target binding in the skull does not significantly affect the diagnostic accuracy in these regions (stage I/II: AUC all subjects, 0.986, 95% CI [0.977–0.996]; AUC w/o high, 0.986, 95% CI [0.977–0.996]). Representative one-year follow-up scans showed that the pattern of retention in these regions is constant over time (Fig. 6b).

## Discussion

Tau PET imaging radiotracers have been proven promising for mapping and tracking AD-associated tau pathology in vivo [4, 6, 22, 41–43] and for differentiating AD patients from controls and individuals with dementia due to other causes [23]. In this study, we have compared the cerebral retention patterns of the most frequently used tracer so far, [^18^F]flortaucipir, with the structurally highly similar tracer [^18^F]RO948 in a head-to-head comparison in cognitively impaired patients with Alzheimer’s disease, MCI due to Alzheimer’s disease, and in cognitively healthy individuals. Across cortical regions, [^18^F]RO948 exhibited overall faster kinetics, reaching peak SUV within approximately 4 min (average 3.93 ± 0.91 min) (Fig. 1b, d, Fig. 2b, d; see also Wong et al [15]), compared with close to 8 min (average 7.94 ± 1.39 min) for [^18^F]flortaucipir (similar to previously published kinetic studies [36, 44–46]).

On visual assessment, [^18^F]RO948 SUVR reached equilibrium earlier than [^18^F]flortaucipir SUVR, which did not plateau during the 100 min course of the scan. The absence of a plateau phase in fully dynamic [^18^F]flortaucipir scans may be problematic for longitudinal assessments if the scanning intervals are not stringently followed, as a delay in scanning time may result in a higher SUVR. Still, both tracers showed linear relationships between SUVR (at 70–90 min for [^18^F]RO948; and 80–100 min for [^18^F]flortaucipir) and Logan DVR values. When comparing SUVR at the indicated time frames, we found that the two tracers bound comparably in neocortical regions (Spearman’s *rho* 0.83–0.97). A slope of 0.84 (95% CI, 0.82–0.85; Y-intercept 0.23 (0.21–0.26)) for the linear regression between the tracers across all neocortical regions ([^18^F]flortaucipir-dependent variable) indicated a slightly higher dynamic range for [^18^F]RO948. [^18^F]RO948 retention in the entorhinal cortex (tau imaging stage I/II) was significantly higher compared with [^18^F]flortaucipir. In the hippocampus, however, retention of [^18^F]flortaucipir was significantly higher than that of [^18^F]RO948, which was shown to at least partially be influenced by the higher [^18^F]flortaucipir off-target signal from the choroid plexus.

Even though the chemical structures of the two tracers are highly similar [9], the level of off-target binding differed substantially, with significantly greater [^18^F]flortaucipir retention in subcortical grey matter structures (thalamus and basal ganglia) as well as in the choroid plexus. In fact, the deep subcortical grey matter structures were the only regions showing significantly higher [^18^F]flortaucipir retention than [^18^F]RO948 on voxelwise group comparisons. The absence of statistical differences in cortical voxels when employing the [^18^F]RO948 > [^18^F]flortaucipir contrast argues for the tracers having comparable neocortical tracer retention, but also argues against off-target binding in the skull/meninges in [^18^F]RO948 scans interfering with cortical retention, at least at a group level. Age-dependent tracer binding in the basal ganglia has previously been reported for [^18^F]flortaucipir [28, 47]. Interestingly, higher basal ganglia retention was observed with increasing age for the two tracers, both within the present study and when compared within the BioFINDER 1 and 2 studies (Fig. 4d). Though regression analysis showed that the slopes were highly similar between the two tracers, the intercepts were clearly different, indicating overall lower affinity of [^18^F]RO948 to off-target sites within these structures, but also low-level age-dependent retention.

The only hitherto published head-to-head in vivo comparison of [^18^F]flortaucipir with another alleged “first generation” tau PET tracer, [^18^F]THK5351, reported overall comparable tracer retention, with [^18^F]flortaucipir exhibiting slightly higher binding in Alzheimer’s disease [48]. However, [^18^F]THK5351 off-target binding was substantially more pronounced. In our study, [^18^F]RO948 showed greater off-target binding in structures outside of the central nervous system such as the skull and meninges. Given the high structural resemblance of [^18^F]RO948 to other “second generation” tau PET tracers [^18^F]PI2620 [17] and GTP-1 [49], it is not surprising that similar off-target binding in meninges and cranial bone have been reported, but also other novel, structurally different tracers such as [^18^F]-MK-6240 appear to exhibit this pattern [10](see also [50] for detailed review). The number of subjects showing high retention of [^18^F]RO948 in the skull or meninges was relatively low (3.4% within the BioFINDER2 study), but in these individuals, off-target binding may interfere with the accurate assessment of cortical signal, at least in smaller adjacent ROIs. To assess the effect of the off-target binding on diagnostic performance, we assessed the area under the receiver operator characteristic curves for AD patients versus controls, with and without subjects with high off-target binding in the BioFINDER2 cohort. We found no significant improvement in diagnostic accuracy after removing the subjects with high off-target binding. There is no clear evidence as to what underlies this off-target binding, however, in follow-up scans in a subsample of our study cohort after 1 year, the pattern of off-target binding in the skull was preserved, indicating that this was not a transient or temporary phenomenon caused by, for example, non-brain penetrant metabolites.

Limitations of this study include the low number of subjects who underwent dynamic scans, the fact that two out of these individuals exhibited very low tracer retention, and the lack of arterial blood sampling to create true input function.

In conclusion, the tau PET tracers [^18^F]RO948 and [^18^F]flortaucipir demonstrate highly comparable retention patterns in neocortical regions. [^18^F]RO948 showed higher SUVR in the entorhinal cortex but lower hippocampal retention, which appeared to be partially due to lower off-target binding in the choroid plexus. [^18^F]RO948 showed favorable kinetic and off-target binding characteristics compared with [^18^F]flortaucipir. Strong off-target signal was seen in the skull with [^18^F]RO948 in a minority of subjects, but that off-target binding did not seem to interfere diagnostic accuracy of the tracer.

## Electronic supplementary material

Supplementary Table 1 (DOCX 14 kb)
