# Supplementary material for: Head-to-head comparison of tau positron emission tomography tracers [18F]flortaucipir and [18F]RO948
Source: Eur J Nucl Med Mol Imaging. 2019 Oct 14;47(2):342–54. doi: 10.1007/s00259-019-04496-0 (PMC6974501; doi:10.1007/s00259-019-04496-0)
Supplement: Supplementary file 1 — (DOCX 14 kb) [file 259_2019_4496_MOESM1_ESM.docx]

**Supplementary table 1. Cerebral and Cerebellar cortex SUV values for ^18^F-Flortaucipir and ^18^F-RO948**

| Diagnosis | ^18^F-Flortaucipir SUV_cerebral cortex_ | ^18^F-RO948 SUV_cerebral cortex_ | ^18^F-Flortaucipir SUV_cerebellar cortex_ | ^18^F-RO948 SUV_cerebellar cortex_ |
| --- | --- | --- | --- | --- |
| AD | 0,864 | 0,472 | 0,491 | 0,257 |
| AD | 0,526 | 0,431 | 0,472 | 0,405 |
| AD | 0,593 | 0,355 | 0,528 | 0,315 |
| AD | 0,517 | 0,330 | 0,481 | 0,323 |
| MCI | 0,718 | 0,503 | 0,647 | 0,426 |
| AD | 0,641 | 0,464 | 0,492 | 0,358 |
| AD | 0,992 | 0,690 | 0,785 | 0,543 |
| AD | 0,754 | 0,576 | 0,618 | 0,471 |
| AD | 0,676 | 0,356 | 0,443 | 0,248 |
| AD | 0,722 | 0,686 | 0,391 | 0,369 |
| AD | 0,874 | 0,675 | 0,533 | 0,408 |
| AD | 0,999 | 0,780 | 0,656 | 0,572 |
| AD | 0,644 | 0,369 | 0,553 | 0,315 |
| AD | 0,798 | 0,426 | 0,719 | 0,408 |
| AD | 0,819 | 0,653 | 0,393 | 0,279 |
| AD | 0,765 | 0,483 | 0,686 | 0,433 |
| MCI | 0,392 | 0,233 | 0,342 | 0,229 |
| AD | 0,680 | 0,449 | 0,409 | 0,285 |
| AD | 0,731 | 0,534 | 0,460 | 0,433 |
| AD | 0,458 | 0,324 | 0,388 | 0,294 |
| CTRL | 0,431 | 0,302 | 0,410 | 0,295 |
| CTRL | 0,567 | 0,358 | 0,506 | 0,341 |
| CTRL | 0,514 | 0,297 | 0,482 | 0,282 |
| CTRL | 0,410 | 0,356 | 0,389 | 0,345 |
| MCI | 0,470 | 0,308 | 0,415 | 0,275 |

Cerebral and cerebellar cortex SUV values for ^18^F-Flortaucipir (80-100 min post injection) and ^18^F-RO948 (70-90 min post injection) in all study participants. AD – Alzheimer’s Disease; CTRL – cognitively normal controls; MCI – mild cognitive impairment; SUV – standardized uptake value (SUV = C_PET (MBq/ml)_ /(Injected Dose_(MBq)_/Body Weight_(g)_)). For the cerebral cortex SUV all cortical FreeSurfer ROIs were used to calculate a global cortical uptake.
